# Aquaporin Function: Seek and You Shall Find!

**DOI:** 10.1093/function/zqaa041

**Published:** 2020-12-21

**Authors:** Dennis Brown

**Affiliations:** Program in Membrane Biology and Division of Nephrology, Massachusetts General Hospital and Harvard Medical School, Boston, MA, USA

Long before Peter Agre et al. discovered the first aquaporin, AQP1, then called CHIP28^1^ it was understood that proteinaceous water channels were involved in rapid, osmotically-driven water movement across biological membranes. Almost 30 years later, I suspect that even he did not anticipate the exponential growth in aquaporin research that has led to the discovery of 13 aquaporins in mammals, and dozens more in species lower on the evolutionary scale, in procaryotes, and in the plant kingdom. More than their sheer number, I wonder if he also foresaw the involvement of these apparently simple water channels in such a vast array of cellular processes, and in the transmembrane movement of a seemingly endless list of small molecules and ions (eg, urea, glycerol, mannitol, sorbitol, ${\text{NH}}_{4}^{+}$, H_2_O_2_, Cl^−^, ${\text{NO}}_{3}^{-}$, arsenite), and even gases^2^ (eg, CO_2_, NH_3_, H_2_S, O_2_, NO). Early studies quickly established a role for some family members (the aquaglyceroporins) in the transport of glycerol and urea in addition to water. But there has been such an expansion of permeability properties of various aquaporins reported over the years that one is left to ask what is the most critical and important physiological role(s) of many of these proteins in the multitude of cell types in which they are expressed. Indeed, some “aqua”porins have a relatively low water permeability, indicating that their principal function lies elsewhere: AQP0, AQP3, AQP6, AQP7, for example, as well as the most divergent members of the mammalian family, AQP11 and 12. The list of aquaporins with “moonlighting” functions—in some cases not obviously related to water permeability—is growing rapidly, leading to still more questions regarding their primary physiological roles. Some cell types even express multiple aquaporin family members in the same membrane. For example, principal cells in some regions of the kidney collecting duct coexpress AQP2, AQP3, and AQP4 in their basolateral plasma membrane—clearly this is not necessary for water permeability alone. Indeed AQP4 knockout has little effect on overall urine concentration, and desert rats—famous for having the most highly developed concentrating mechanism in the mammalian kingdom—do not express AQP4 at all in their kidneys^3^! In contrast, many studies have confirmed the critical role of AQP2 in water reabsorption by the kidney^4^; loss of function mutations in AQP2 causes autosomal nephrogenic diabetes insipidus—a loss of urinary concentrating capacity.

In many cases, the elusive role of many aquaporins in normal tissue and organ physiology has not been specifically determined, even by the use of AQP knockout mice, which often (but not always) show no or minor phenotypes. However, an increasing number of studies both in vivo and in vitro implicate aquaporins in normal physiology and disease states that have often not been experimentally correlated with their channel activity. For example, aquaporins are involved in events such as cell migration, epithelial, and organ development, obesity, inflammation, cancer progression, and various neurodegenerative diseases, including Alzheimer’s disease. Facilitating water and solute movement across membranes might indeed be involved in these processes, but other features of the aquaporin proteins could also play an important role—such as their participation in protein–protein interactions with components of the cytoskeleton, as well as with various signal transduction mechanisms and other intracellular pathways.^5^ Understanding the physiological importance of aquaporin function is, therefore, an ongoing quest; the enormity of the task is highlighted by just a few disparate examples selected from the most recent literature:

## Aquaporin 1 and Neural Crest Cell Migration

AQP1 was the first aquaporin to be implicated in cell migration and angiogenesis. Knockout of AQP1 in mice not only leads to slower tumor development and lower mortality, but also can result in slower wound healing.^6^ This migratory role extends to chick neural crest cells as they progress through the extracellular matrix to perform their critical function in development.^7^ AQP1 stabilizes cell processes (filopodia) and stimulates their extension and migration via interaction with focal adhesion kinases, EphB guidance receptors, and by stimulating metalloproteases in the matrix. While suspected, the role of water transport by AQP1 in these events was not directly examined, but these data certainly highlight the involvement of AQP1 in a process that would not be immediately predicted based on channel activity alone.

## Aquaporin 4 and Central Nervous System Edema

The predominant aquaporin expressed in the brain is AQP4, normally polarized on the foot processes of astrocytes that wrap around capillaries, where it is engaged in fluid exchange, as well as K^+^ buffering and calcium signaling. There has been considerable interest in AQP4 in the clearance from the brain of proteins including β-amyloid and tau as part of the so-called glymphatic system, a name derived from the combined role of glial cells and the lymphatic system in brain fluid management. While AQP4 function in the brain is most often linked to its water permeability, its predisposition and capacity for self-assembly and adhesion in the pathophysiology of neurodegenerative diseases remains controversial. However, a recent study implicates increased AQP4 expression at the plasma membrane of astrocytes in the central nervous system (CNS) in hypoxia-induced edema.^8^ Importantly, this translocation process depends on phosphorylation of AQP4 by calmodulin. Critically, inhibition of calmodulin with trifluoperazine significantly reduced plasma membrane AQP4 expression and CNS edema, and enhanced recovery in this rat model of CNS damage. Thus, the authors suggest that calmodulin inhibitors may be a therapeutic strategy for the millions of individuals who suffer from trauma-induced CNS edema, by preventing increased water flow into the CNS through AQP4.

## Aquaporin 3 Inhibition Reduces Inflammatory Liver Injury

AQP3 is permeable to H_2_O_2_, and has been implicated in promoting tissue inflammation by increasing reactive oxygen species in expressing cells, such as macrophages, and stimulating damage processes such as fibrosis.^9^ Searching for therapeutically useful specific aquaporin inhibitors has been an ongoing (and not particularly successful) process for many years, but now a new monoclonal antibody that blocks AQP3 permeability to H_2_O_2_ in liver macrophages is reported to reduce CCl_4_-induced liver injury: it does this by inhibiting NF-κB-mediated signaling and macrophage activation in the liver.^10^ The hope is that blocking AQP3 permeability to H_2_O_2_ may be more generally applicable to other inflammatory processes involving AQP3-expressing macrophages.

From just this small sampling of the recent literature on aquaporins, it is apparent that their involvement in (patho)physiological processes is widespread and often unexpected. In addition to their broad range of permeability properties, their capacity to interact with a host of other cellular proteins—from structural and cytoskeletal elements, through signaling and trafficking components, to other membrane channels and transporters—indicates that we have not yet discovered the full extent to which they affect our physiological well-being. How much more do we have to learn about this remarkable and versatile family of proteins? Only time and effort will tell. The subtitle of this opinion piece “seek and you shall find” is intended to encourage yet more researchers in different fields to contribute to this exciting endeavor.

## Conflict of Interest Statement

The author has no conflicts of interest to declare.

## Funding

The author acknowledges support from the National Institutes of Health for his own work on aquaporins, currently through grant number 5RO1 DK096586-09.
